# Evaluation of the Cochrane Consumers and Communication Group’s systematic review priority-setting project

**DOI:** 10.1186/s12961-020-00604-x

**Published:** 2020-09-02

**Authors:** Anneliese Synnot, Allison Tong, Rebecca Ryan, Sophie Hill

**Affiliations:** 1 Centre for Health Communication and Participation, School of Psychology and Public Health, La Trobe University, Melbourne, Victoria Australia; 2 Cochrane Australia, School of Public Health and Preventive Medicine, Monash University, Melbourne, Victoria Australia; 3 Sydney School of Public Health, The University of Sydney, Sydney, NSW Australia; 4 Centre for Kidney Research, The Children’s Hospital at Westmead, Westmead, NSW Australia

**Keywords:** Research priority-setting, evaluation, systematic review

## Abstract

**Background:**

Health researchers and funders are increasingly consulting with stakeholders to set their research agendas but these activities are rarely evaluated. The Cochrane Consumers and Communication Group (CCCG) conducted a priority-setting project for systematic reviews in partnership with stakeholders (consumers/patients, health professionals, policy-makers and others). In this paper, we aim to describe our evaluation of the project’s processes and outcomes.

**Methods:**

We used a 10-element conceptual framework designed to evaluate processes (e.g. stakeholder engagement, use of explicit process) and outcomes (e.g. improved decision-making quality, stakeholder acceptance and understanding) of health priority-setting. Data sources included empirical data (feedback surveys, project documents and CCCG editorial policies) and CCCG staff reflections. Data were analysed using content analysis.

**Results:**

The project met three and partially met two of the process elements, for example, by engaging key stakeholders throughout the project and using pre-determined and transparent methods that offered multiple and meaningful ways to contribute. The project met three and partially met two of the outcome elements. Stakeholders were satisfied with and accepted the process and an additional six Cochrane Review titles aligned with stakeholder priorities are now being conducted in partnership with stakeholders. The project has also directly influenced the editorial work of CCCG, for example, by shifting its organisational focus towards coproduction, and indirectly influenced the work of Cochrane’s prioritisation and coproduction activities. Some areas were identified as having room for improvement, for example, there was low participation by people from diverse backgrounds, stakeholders could contribute to most but not all project stages, and there was no formal way for stakeholders to appeal decisions at project end. In the 3 years since its completion, the Cochrane Reviews are nearing completion but none of the reviews have been published.

**Conclusion:**

We demonstrated that our priority-setting methods were broadly in line with best practice and the project resulted in many positive outcomes beyond just identifying the top priorities for research. Our evaluation framework and recommendations for future evaluations may be of use to priority-setting researchers planning similar activities.

## Background

Health researchers and funders internationally are increasingly consulting with stakeholders to set their research agendas [12, 22]. This can increase the relevance of research to meet the needs of those it affects, while ensuring judicious use of increasingly scarce resources and avoiding research waste [6, 12]. The stakeholders involved include the public, patients, carers and their representatives (collectively ‘consumers’), plus health professionals, policy-makers and other health decision-makers [11].

As research priority-setting has gained traction, so too have the calls for its evaluation [1, 20, 33]. Evaluating research priority-setting is challenging as there are no frameworks specifically designed for this task [23, 33] and no consensus about the purpose of such evaluations nor of what we should evaluate or how [1, 33]. Further, there are very few published examples of research priority-setting evaluations [1, 14]. Existing evaluations tend to focus on priority-setting processes and/or the quality of the stakeholder engagement [3, 19] rather than on outcomes or impacts [1, 20, 33].

Cochrane is a leader in the production and publication of systematic reviews and currently encourages its Groups and Networks to undertake prioritisation exercises with stakeholders to shape their systematic review topics [10]. Monitoring and evaluation is a recommended component of systematic review prioritisation but there are few published examples [10, 14, 23, 24].

Given the proliferation of research priority-setting exercises and the near absence of accompanying evaluations, we present our evaluation of the systematic review priority-setting project conducted by the Cochrane Consumers and Communication Group (CCCG) [31, 32]. In this paper, our aim is to describe our evaluation of the processes and outcomes of the project. Such an evaluation can inform future CCCG priority-setting exercises but can also be used to guide others’ priority-setting activities or provide a template for evaluation.

## Methods

### Context: the CCCG priority-setting project

The CCCG is located in Melbourne, Australia, within the Centre for Health Communication and Participation (the ‘Centre’), La Trobe University. The CCCG coordinates the publication of Cochrane systematic reviews (Cochrane Reviews) of “*interventions that affect the way people interact with healthcare professionals, services and researchers*” [9]. The CCCG priority-setting project was conducted in Australia in 2015–2016, with the aim of identifying five future Cochrane Review intervention topics within the CCCG’s scope. We defined this as “*activities that help patients, consumers and carers to be knowledgeable about their health and to participate in their health in different ways. This includes being able to express their views and beliefs, make informed choices, and access high quality health information and health services*” [26].

The project was overseen by an 11-member steering group, including consumers, health policy-makers, health professionals, people from health services, research funders and researchers. The first stage consisted of an international online survey (with 151 respondents) to generate ideas for research [31]. The second stage involved a 1-day workshop with 28 stakeholders to select and refine five broad topics for future Cochrane Reviews. The final stage involved a mapping exercise to arrive at five specific priority Cochrane Reviews [32].

The purpose of our post-hoc evaluation was threefold, namely to capture the range of ways we saw the priority-setting project influencing the work of the CCCG, to reflect on our practice, and to inform how we might go about such an activity again.

### Evaluation framework

We used a 10-element conceptual framework devised to evaluate success in priority-setting in healthcare systems [28]. While it was not specifically designed to evaluate priority-setting in research, it has previously been adapted to evaluate Cochrane Review priority-setting exercises [23, 24] and it was developed with considerable stakeholder input. We used the framework's descriptions of the five process and five outcome elements to devise 27 questions to guide the evaluation. We integrated relevant equity considerations using the questions devised by Nasser [23] into our consideration of the elements used by Sibbald et al. [28] The process evaluation (Table 1) and the outcome evaluation (Table 2) along with the data sources used for each element have been provided.

**Table 1:** Framework for process evaluation: elements, questions and data sources

Element [28]	Questions	Data sources
Stakeholder engagement	1. Were key stakeholders who might be affected by the choice of review topics, or seek to use the reviews (such as consumers, health professionals and decision-makers) involved effectively in the decision-making process?	- Project documents (workshop materials, steering group meeting minutes)
	2. Were multiple techniques were used to identify stakeholders?	- Workshop feedback survey
	3. Were stakeholders offered multiple ways to contribute?	- Communication materials (final report, publications)
	4. Was CCCG committed to genuine engagement through partnership and empowerment?	
	5. Were stakeholders satisfied with their level of involvement in the decision-making process?	
Use of explicit process	6. Was the priority-setting process pre-determined and made transparent to stakeholders?	- Project documents (survey and workshop materials)
	7. Were internal and external stakeholders probed for information relevant to priority-setting decisions?	- Communication materials (project webpage, final report)
	8. Were the methods used to set priorities understandable, transparent and relevant for different stakeholders?	
	9. Was communication with stakeholders well-coordinated, systematic and well-planned?	- Workshop feedback survey
	10. Was information about the project communicated effectively using multiple vehicles?	
Information management	11. Was information used to set priorities, including sources used and how it was collected and collated, made explicit to those setting priorities? Was it deemed sufficient?	- Project documents (survey and workshop materials, steering group meeting minutes)
		- Communication materials (publications, project webpage)
Consideration of values and context	12. Was the mission, vision and values of CCCG used to guide priority-setting decisions, and made explicit?	- Centre 2019 Year in Review Report
	13. Did priority-setting decisions consider CCCG’s strategic directions as a unit?	- Project documents (steering group meeting minutes, project plan)
	14. Were the (potential) values of stakeholders, both those involved in the project and those not involved, used to guide priority-setting decisions?	
		- Communication materials (publications, project webpage)
Revision or appeals mechanism	15. Was there a formal mechanism for reviewing decisions, whereby stakeholders could identify failures and errors or contribute new information?	- Project documents (workshop materials, steering group meeting minutes)

*CCCG* Cochrane Consumers and Communication Group

**Table 2:** Framework for outcome evaluation: elements, questions and data sources

Element [28]	Questions	Data sources
Improved stakeholder understanding	1. Did stakeholders obtain more than a knowledge of the priority-setting process, but gained insight into broader aspects of priority-setting (e.g. the rationale for priority-setting generally) and/or the CCCG/Cochrane (e.g. its mission, values)?	- Workshop feedback survey
		- Project documents (communication with stakeholders)
		- CCCG Editorial team reflections
Shifted priorities and/or reallocation of resources	2. Were more Cochrane Reviews topics selected that were relevant to stakeholders?	- CCCG Editorial team reflections
	3. Did Cochrane Review topics that reflect the priorities of stakeholders get funded and conducted?	- Communication material (publication)
Improved decision-making quality	4. Were CCCG decisions and strategic direction more consistent with the priorities generated?	- CCCG Editorial team reflections
	5. (Did future CCCG priority-setting activities aim to build on earlier efforts?)^a^	- CCCG Editorial documents and policies for review production
Stakeholder acceptance and satisfaction	6. Did stakeholders express satisfaction with the process?	- Workshop feedback survey
	7. Did stakeholders partner with researchers to conduct the priority Cochrane Reviews?	- Informal feedback from stakeholders
	8. (Did stakeholders use the results of the priority reviews?)^a^	- Priority review production metrics
Positive externalities	9. Were the results of the priority-setting process shared widely?	- Project documents
	10. Did research funders and research institutes include the priorities as part of their research agenda or strategic planning?	- Communication materials (webpage, final report, publications)
	11. Was the priority-setting process and/or its results emulated by or did it influence the work of other organisations?	
	12. (Did the priority reviews result in changed policies, legislation or clinical practice?)^a^	- CCCG Editorial team reflections

*CCCG* Cochrane Consumers and Communication Group

^a^Question unable to be assessed because the priority reviews selected in the priority-setting process are not yet published. The question is included for completeness

### Data collection

Data included a combination of empirical data collected during and after the priority-setting project as well as project and editorial team reflections collected after the project. Empirical data included workshop feedback surveys and project documents. The feedback surveys were intended to be used in the event of future workshops being run by CCCG and were completed by 25 of the 28 stakeholders who attended the prioritisation workshop. Questions probed the degree to which stakeholders felt able to participate, with open-ended questions about what worked well and what could be improved. Responses to the feedback survey had been previously entered into a spreadsheet, with free-text responses grouped into similar concepts (Additional file 1). Project documents included the project plan, steering group meeting documents and minutes, recruitment materials, email correspondence between stakeholders and the project team, online survey materials (see online appendices of Synnot et al. [31]), workshop materials (see online appendices of Synnot et al. [32]), the project webpage (including archived versions) [4], the final report [30], and academic publications [31, 32].

Post-project empirical data included CCCG documents, such as publicly available author resources and editorial policies [8] produced after 2016 (when the priority-setting project was completed) and the Centre’s Year in Review Report [5]. It also included descriptive data about CCCG’s Cochrane Review portfolio post-2016 (such as protocols and reviews published and reviews that included stakeholder engagement). Finally, CCCG editorial team reflections were collected in a structured feedback session held 18 months after the project was completed. CCCG editorial staff were provided with the evaluation framework and provisional results and invited to reflect on these and to add further information and examples.

### Analysis

One researcher (AS) undertook a content analysis, identifying and grouping information in the various documents according to the questions in our evaluation framework [25]. Each document was reviewed against the 27 evaluation questions grouped into 10 elements using an Excel spreadsheet. We made notes about, or from, the different data sources in the appropriate cells of the spreadsheet. Notes could be made about the document (e.g. ‘available on the project webpage’ or ‘stakeholders given the option of contributing online or by phone’) or made using information within the document (e.g. direct quotes). We then grouped all notes by evaluation question and then by evaluation element, writing a summary of the data for each question and then for each element. See Additional File 2 for tabulated examples of the raw data that contributed to each question within the 10 elements of the framework.

## Results

### Evaluation of priority-setting processes

#### Stakeholder engagement

All key stakeholders were involved in the decision-making process. Consumers and consumer group representatives, health policy-makers, health professionals, health service staff and research funders were all included in the steering group (*n* = 11), the online survey (*n* = 151) and the workshop (*n* = 28). The steering group was involved in the decision-making process by influencing key directions in the methods used, whereas workshop participants were involved in decisions by voting on the top research priorities to go forward as Cochrane Reviews. However, as steering group members and workshop participants pointed out, participation in the online survey by Indigenous people and people from culturally and linguistically diverse backgrounds was low. We subsequently used a sampling frame to guide workshop recruitment and approached specific organisations representing these groups in the recruitment process, but participation rates in the workshop remained low (3/28 people from culturally and linguistically diverse backgrounds and 1/28 Indigenous people).

Across the project, we used multiple techniques to identify stakeholders and offered stakeholders multiple ways to contribute. We recruited stakeholders for the survey and workshop using networks of the project team and steering group. For the online survey and workshop, we also contacted a range of health organisations, inviting their members to forward an email invitation to participate. One steering group member encouraged face-to-face methods for the online survey to facilitate participation for particular groups, such as older people, but this was discounted on account of limited resources. To make participation more accessible, online survey participants could, on request, take part by phone or post (2/151 people accepted this offer).

We were committed to genuine engagement at the outset, following the principles of coproduction [16]. Examples of how this was operationalised included partnering with stakeholders in all project stages, giving the steering group decision-making powers and using multiple strategies in the workshop to facilitate true participation. These workshop strategies included having 50% consumer participation (mitigating power imbalances) and reimbursing time and transport costs. One steering group member said, “*I appreciated the respectful and rigorous way in which consumer perspectives were sought and incorporated in this project*”.

All workshop participants agreed with the statements “*I feel that my contribution was valued and heard*” and “*the materials and resources used during the day helped me understand my role and make a contribution*”, which suggests that they were satisfied with their participation. In addition, of the 64 free-text comments received in the workshop feedback survey, 43 included positive feedback and 21 offered suggestions for improvement. Of these, 5/25 workshop participants suggested that the workshop structure may not have allowed quieter participants to contribute and 3/25 suggested the time constraints limited discussion.

#### Use of explicit process

The priority-setting methods were pre-determined and made transparent to stakeholders as they were planned prior to the first steering group meeting and made available on the project webpage. In addition, participants in the online survey and workshop were provided with information about the CCCG along with a summary of the methods and how their contribution would be included in project. With respect to including internal and external stakeholders, we did not specifically seek to include CCCG staff and editors but instead focussed on probing external stakeholders for their research priorities.

The methods used to set priorities in this project were understandable, transparent and relevant for different stakeholders. The steering group reviewed and approved our methods and all workshop participants surveyed agreed with the statements that the information they received helped them understand what was expected of them before they came, and that the materials and resources used on the day helped them understand their role and make a contribution. A workshop participant stated in the final report, “*thank you so much for all your help to allow me to participate in the workshop. It was one of the best experiences of my life, I felt ‘heard’ and I hope I was able to help in some way*”.

Communication with stakeholders was well coordinated, systematic and well planned. The steering group were given agendas and meeting documents approximately a week prior to meetings and the minutes were circulated within a week. Similarly, workshop participants were provided with a pre-reading pack several days prior to the workshop (see online appendices in Synnot et al. [32]) while online survey reminders were sent weekly to all email addresses on the recruitment list. During the project, information was communicated using the project webpage [4] and at project completion with a professionally formatted final report [30] and in academic publications [31, 32].

#### Information management

The primary information used to determine the five priority Cochrane Reviews (namely the online survey and the workshop) was pre-determined and made explicit to all stakeholders throughout the project. However, the intended final step, generating evidence maps of the priority topics for final selection by the steering group, was not undertaken because the complexity of the CCCG scope and the broad categories of interventions within many areas makes this task too abstract and not helpful for editorial decision-making in terms of specific topics for reviews. In its place, the project team undertook a more pragmatic step of mapping the priority topics selected at the workshop against the CCCG review portfolio and then sought steering group and editorial approval. While the steering group endorsed the new approach, the final step in the priority-setting process involved no stakeholders in the decision-making.

#### Consideration of values and context

The Centre’s mission (where CCCG is located), is to conduct research to “*to improve people’s health and wellbeing through the generation and promotion of evidence-informed strategies for consumer communication and participation in health* [and] *to strengthen the active involvement of consumers in health care, policy and research*” [5]. This mission was included in the project plan which was shared with the steering group and made available on the project webpage. It also informed the methods used, specifically giving stakeholders the opportunity to shape, identify and select the priority topics. The final selection of five priority Cochrane Reviews was informed by a set of editorial criteria reflecting CCCG’s more review-specific priorities (for example, that the review could be completed in a timely manner). One of these review-specific criteria was that the author team was agreeable to consumer/stakeholder engagement in their review. It was included because it was a priority research topic identified by stakeholders in the survey and workshop, reflecting the way in which stakeholders’ values were included in the decision-making process.

#### Revision or appeals mechanism

There were two ways in which decisions were formally reviewed by stakeholders. Steering group meetings provided three opportunities for members to review and contribute to project decisions. For example, at one meeting, members urged further recruitment efforts to increase the diversity of participants in the online survey. We also gave workshop participants an opportunity to refine and contribute new ideas to the priorities generated in the online survey. As a result, one new priority topic was added to the list of priorities, which were voted on at a later stage in the workshop. However, once the five priority topics were announced, we had no formal appeals mechanism for stakeholders.

### Evaluation of priority-setting outcomes

#### Improved stakeholder understanding

Based on the workshop feedback survey, stakeholders understood the nature of the priority-setting process since they all agreed with the statements that they understood what was expected of them and felt able to make a contribution. In addition, at least six workshop participants left feedback or contacted the team after the workshop to express their interest in being involved in the priority Cochrane Reviews, which suggests they had gained insight into ways of being involved in CCCG/Cochrane and understood what they were contributing to. Stakeholders in the steering group also demonstrated improved understanding given one steering group member subsequently initiated a priority-setting exercise in her workplace and another became a co-author on one of the priority Cochrane Reviews.

#### Shifted priorities and/or reallocation of resources

At project completion, the five Cochrane Review titles that were selected as priority topics directly reflected the top priority areas as identified and voted on by stakeholders. One of the priority titles [13], an existing Cochrane Review needing updating, later became two protocols for new reviews as a result of a new coproduction process with stakeholders [18, 21]. In response to stakeholders’ desire to be involved in the priority Cochrane Reviews and to reflect the degree to which consumer engagement in health was a priority topic, CCCG required the priority review author teams to involve stakeholders. To support the timely publication of the priority reviews, the CCCG reallocated editorial resources to provide additional guidance and support to the priority review teams, for example, providing rapid assistance with methodological queries and prioritising search development. In addition, the CCCG sought and received funding to support the production of one review conducted by the CCCG editorial team and one review conducted by an external author team. Despite these achievements, more than 3 years after the prioritisation process (at the time of writing) no reviews have yet been published (all reviews have published protocols, two reviews have been submitted for editorial assessment, one rejected (for reasons unrelated to the engagement process), and the remainder should be completed within 2020).

#### Improved decision-making quality

The priority-setting project resulted in a shift in CCCGs editorial policies and its organisational values. For example, the CCCG changed its editorial policy on title proposals and updates, requesting they align with stakeholder priorities, with more invited or commissioned title proposals. With regards to its organisational values, since the priority-setting project, CCCG now conducts its in-house reviews with stakeholder input, with one co-produced review and one with stakeholder input now underway. Subsequently, the involvement of three editorial staff in prioritisation and coproduction activities led to the initiation of a coproduction network for Australian researchers and consumers, and two new collaborations with international researchers involved in stakeholder engagement activities.

#### Stakeholder acceptance and satisfaction

Stakeholders across the project stages expressed satisfaction with the process by showing a willingness to contribute to subsequent project stages. For example, at least one participant took part in the online survey and then accepted an invitation to participate in the workshop. Five workshop participants and steering group members accepted an invitation to contribute to the final report and five joined author teams or stakeholder panels for the priority reviews. In addition, workshop participants gave overwhelmingly positive comments in the feedback survey, with 14/25 participants praising the structure and/or facilitation of the day, with typical comments including a “*great day*” that was well planned/communicated/organised.

All the priority reviews involve stakeholders in sometimes novel ways. In some reviews, there are consumer co-authors and others have a stakeholder advisory group. One of the priority reviews involved stakeholders in the peer review process, with a group of health policy-makers being briefed on the review in its early stages and subsequently providing peer review of the protocol.

#### Positive externalities

The results of the priority-setting project were shared with stakeholders via a professionally designed (and coproduced) reports and promoted via health-sector aimed newsletters, blogs, policy-maker seminars and meetings, and academic presentations and publications.

While we are not aware of research funders or research institutes including the priorities as part of their research agenda or strategic planning, it has influenced the work of others. For example, an Australian state health department replicated our methods to inform the development of their state-wide policy on consumer engagement [17]. In addition, the project’s findings have influenced other systematic reviewers, for example, Selman [27].

The priority-setting project has also indirectly influenced the work of Cochrane. As a result of this project, CCCG staff contributed to Cochrane’s guidance on priority-setting [10], with the CCCG priority-setting project described in considerable detail as an example of good practice. Finally, CCCG successfully advocated for a change to Cochrane’s now discontinued priority review funding programme, whereby it allowed a longer funding period for complex reviews such as those being produced with stakeholders.

## Discussion

The CCCG priority-setting project met 6 and partially met 4 of the 10 process and outcome elements in the Sibbald et al. [28] framework. With respect to the priority-setting process, the elements of Stakeholder engagement, Use of explicit process, and Consideration of values and context were met with the remaining two (Information management and Revision or appeals mechanism) partially met. All key stakeholder groups were represented, and they were offered multiple and meaningful ways to contribute. The methods used were pre-determined and transparent and supported by a well-coordinated and multi-pronged communication strategy. Most of the information used to set priorities was made clear to stakeholders and the priority-setting decisions considered CCCG’s mission/strategic direction and stakeholder values. Finally, stakeholders could review and contribute to decisions made at some but not all points in the project. The process evaluation demonstrated the methods we used were broadly reflective of best practice in priority-setting.

For the outcome evaluation, the project met three elements (Improved stakeholder understanding, Improved decision-making quality, and Stakeholder acceptance and satisfaction) and partially met two (Shifted priorities and/or reallocation of resources and Positive externalities). Stakeholders gained insight into the broader aspects of priority-setting and CCCG/Cochrane. The project resulted in more Cochrane Review titles that were relevant to stakeholders, with re-allocated resources within CCCG and some external funding secured. CCCG also amended some editorial policies to better reflect stakeholder priorities and shifted its organisational values, with a greater focus on co-production in its work and new collaborations. Stakeholders demonstrated their acceptance and satisfaction with the process and all priority reviews now involve stakeholders. While no research funders or research institutes included the priorities as part of their research agenda, the methods used have been replicated by a state health department and have indirectly influenced the work of Cochrane. The outcome evaluation demonstrated that priority-setting projects can result in many more outcomes than just identifying the top research priorities.

We identified some areas for improvement and make a number of suggestions that could be addressed in future projects. With regards to processes, there was low participation by people from diverse backgrounds, stakeholders were not involved in the final decision-making step, and there was no formal mechanism for stakeholders to review the final priorities selected. We suggest partnering with organisations representing diverse groups early in the process and ensuring there are sufficient time and resources to offer multiple ways for people to contribute. Priority-setting projects like ours, which generate broadly scoped priorities, may benefit from doing more of the conceptual sorting of priorities before the final opportunity for stakeholder input. Alternatively, a final stakeholder workshop or two-stage online voting round would allow stakeholders (including people from diverse backgrounds) to be involved in the final prioritisation step and provide a formal mechanism for stakeholders to review the priorities selected.

Two limitations of this evaluation are found in its design. First, it was planned and undertaken sometime after the project was completed, meaning some information and context may have been lost over time. While it did allow for some outcomes to develop (such as the impact on CCCGs organisational values and focus on coproduction of its reviews), the evaluation would have been strengthened with a prospective approach, with interviews with stakeholders (rather than solely editorial staff reflections) during and after the project. Second, the evaluation was undertaken by members of the priority-setting project team and CCCG editorial team, which may have introduced a positive bias.

There are few evaluations of priority-setting for systematic reviews with which to compare our results (Fadlallahet al. In press). With regards to process evaluations, Nasser et al. [24] assessed the priority-setting processes of 13 Cochrane groups. They found that most groups involved practitioners in the decision-making process and made attempts to make their process transparent to stakeholders. Only one group had specific strategies to develop fair and inclusive partnerships and one had an appeals mechanism. Our priority-setting process, with considerable stakeholder engagement, in particular consumers, and using comprehensive and explicit methods compares favourably against these. With regards to outcome evaluations, Buckley [2] and Christie [7] both assessed the research activity (including systematic reviews) that had arisen subsequent to their stakeholder-derived research priorities. Compared to our evaluation, these would answer just one of the elements (Shifted priorities and/or reallocation of resources) that we report against.

There are two outcomes in our evaluation that are worthy of further discussion. First, it is taking significant time for the priority reviews to be completed. Such delays are not uncommon given that large, complex reviews are typical with CCCG reviews, with teams engaging stakeholders and working on reviews largely in unfunded time. If following standard editorial procedures and without a dedicated funding stream for priority research, Cochrane editorial groups and stakeholders should be aware that the prioritised reviews may take some time to come to fruition. However, we note that, at the time of writing, Cochrane has a portfolio of prioritised reviews in response to COVID-19 and, due to the emergency, the expected timeline for these reviews is between 6 weeks and 6 months [15]. Future approaches to producing prioritised reviews, therefore, may expedite their production. Second, the project resulted in editorial and organisational shifts within CCCG and indirectly influenced the work of Cochrane. These kinds of outcomes are not uncommon in research priority-setting partnerships, Staley and Crowe [29] refer to them as “*collateral benefits*”. However, they are not captured in existing outcome evaluations for systematic review priority-setting exercises [2, 7].

This evaluation highlights the need for further work on a framework to evaluate research priority-setting and, in particular, its outcomes. The framework we used [28] was designed for priority-setting in healthcare systems and it was hard to apply some of the outcome elements to our context. The Viergever et al. [34] framework was designed to guide good practice in health research priority-setting and has been used for evaluations [19], but it does not extend to outcomes. Future work that includes both stakeholders’ views and key themes from the literature to generate a framework for evaluating processes and outcomes of research priority-setting would be helpful.

Based on our experience, we make a number of recommendations for those wishing to evaluate systematic review priority-setting exercises. Future evaluations could use the Sibbald et al. [28]conceptual framework along with our 27 questions. A prospective evaluation approach would allow interviews or surveys to be undertaken with stakeholders, the project team and project funders (if relevant) during and after the project. Detailed records of project decisions and meetings should be kept as an additional data source to provide examples of how certain questions were met or not (e.g. was there a commitment to genuine engagement through partnership and empowerment?). Some of the questions relating to outcomes (e.g. did stakeholders use the results of the priority reviews?) are dependent on the priority reviews being conducted and then published, so further data collection could be undertaken after the outputs of a priority-setting exercise are produced. A further evaluative step could include an analysis of the impact of any reviews in terms of policy or practice but this would require a much longer timescale.

## Conclusions

We have built upon an existing conceptual framework and used easily replicable methods to conduct one of the first evaluations of the processes and outcomes of a priority-setting exercise for systematic reviews. The process evaluation demonstrated that the methods we used were broadly in line with recommended practice, including inclusive stakeholder engagement, use of explicit processes, and the consideration of values and context. It also identified areas for improvement, such as increasing participation by people from diverse backgrounds and ensuring that stakeholders can contribute to all stages of the process. These findings broadly endorse the methods we used, giving confidence to those who want to replicate or use similar methods.

The outcome evaluation demonstrated the ways the project improved stakeholder understanding and acceptance (e.g. stakeholders involved in the project accepted invitations to be involved in the priority reviews), decision-making quality (e.g. the CCCG now has greater focus on coproduction of its reviews) and brought about a range of positive externalities (e.g. influencing the work of an Australian health department). Many of the benefits we identified have not been acknowledged previously, and clearly show that priority-setting exercises can produce a range of ‘collateral’ benefits to the organisation and others beyond just identifying the top priorities for research.

Future evaluations of research priority-setting exercises could use the Sibbald et al. [28] conceptual framework, along with the questions we devised, to undertake a prospective evaluation using both project documents and interviews or surveys with stakeholders, the project team and funders. For a complete picture of the outcomes, final data collection should take place sometime after the project is completed.

## Supplementary information

**Additional File 1.** Results of workshop feedback survey

**Additional file 2.** Evaluation raw data

## Data Availability

Much of the data used in this study is publicly accessible and has been appropriately referenced or are available in its supplementary material. Other data used in the current study are available from the corresponding author on reasonable request.

## Abbreviations

CCCG: Cochrane Consumers and Communication Group
