# Supplementary material for: Evaluation of the Cochrane Consumers and Communication Group’s systematic review priority-setting project
Source: Health Res Policy Syst. 2020 Sep 2;18:98. doi: 10.1186/s12961-020-00604-x (PMC7465879; doi:10.1186/s12961-020-00604-x)
Supplement: Supplementary file 1 — Additional File 1. Results of workshop feedback survey [file 12961_2020_604_MOESM1_ESM.docx]

**Additional File 1. Results of workshop feedback survey**

From: Synnot et al. Evaluation of the Cochrane Consumers and Communication Group’s systematic review priority setting project

N = 25/28 workshop attendees completed the survey

**Question 1: Please consider the following statements and tick a response option**

|  | **Agree** | **Neutral** | **Disagree** |
| --- | --- | --- | --- |
| The information I received before coming helped me understand what was expected of me before I came | 25 (100%) | 0 | 0 |
| The materials and resources used during the day helped me understand my role and make a contribution | 25 (100%) | 0 | 0 |
| I felt that my contribution was heard and valued | 25 (100%) | 0 | 0 |

**Question 2: What do you feel worked well today?**

Structure of the day

1. the small groups to brainstorm/expand on the topics; the stickers/voting to reduce the list of topics; the proportion of participants meant it was a broad/balanced discussion
2. Limited breaking in between activities; breakout tables; rotating groups/topics; time to pick 5 topics; recording information as we went on iPad/screen
3. I think having a structured day worked really well; allowed us to focus on the task at hand; but still allowed participants to contribute.
4. clear structure and timeframes; clear summaries of key points provided by lead facilitator; good balance of perspectives and experiences in the room
5. Looking at the issues and seeing if any were missed; the group discussions and feedback that resulted in highlighting other issues; being able to expand on the topics and introducing new ones such as transition
6. Breaking into smaller groups was useful; overheads; written information handed out/emailed before the day and on the day; hearing the perspective of others
7. Running to time; clear instructions before each session; opportunities to be heard; knowledgeable facilitators; good directions; effectively carried out with such a diverse group
8. System of exploring each issue before prioritising them. Breaking into small discussion groups allowed everyone to be heard.
9. Explanation of the purpose of the workshop; explanation of the plan for the day; participant contribution; small group discussions; Great day!!! Thank you for giving me the opportunity to participate. I feel heard.

Facilitation

1. Facilitation was excellent. Kept things on track.
2. The team! Great work.
3. Great facilitation and team work by Cochrane group; good range of opinions and view points.
4. The small group discussion; Excellent facilitation; material provided in preparation for the workshop.
5. Excellent facilitation; well organised and allowed time for discussion

Clear explanations

1. Explanation then discussion immediately put on a screen; moving to different tables worked well; documentation excellent.
2. Good participation level; well co-ordinated; goodwill
3. Information was explained well
4. Lunch. Information was communicated well

Small (and large) group discussions

1. Small group discussions; voting process; food :)
2. Setting the context; small table discussions; priority setting
3. Small group worked allowed me to explain my point of view and perspective as it related to specific topics
4. small groups
5. Large group discussions to open new topics
6. Opportunity for discussions about a range of issues of interest.

Other

1. My experience as a consumer of medical health system and a consumer representative at [health service] has given me some understanding of these issues, today's discussion offered of the context of my understanding
2. The overall consultative nature of the event. The fact that not too many people were present meant we could all contribute. The suitability of physical resources like pen, paper, tables etc.

**Question 3: What do you feel could’ve been improved?**

Finding ways for ‘quieter’ participants to contribute

1. How to make people speak up in a largish space
2. Some people were left to talk all the time. This happened when everyone was asked "If they had any questions?" Some people like the sound of their own voice (Sorry). Whilst in the group before lunch people started saying which number was good. I felt they were trying to influence others to put their red dots on specific issues. This was being unfair.
3. Reflections and discussions - mid morning. Was a little difficult for 'quieter' participants to get a word in or to add new reflections or discussion points. The session was interesting but difficult to add to at times.
4. Some people didn't speak - maybe more opportunity to hear from others would be good.
5. Maybe 3 separate lead-up workshops; 1) professionals, 2) consumers, 3) carers. Then follow-up day afterwards. I feel that the survey did not represent the voices of the disadvantaged, marginalised group of carers/consumers who are just surviving and can barely put one foot in from of the other. There are 700,000 carers in Victoria alone.

Time constraints restricted discussion

1. Think there needed to be more time for participants to identify missing issues- maybe allowing an opportunity to write them out and submit might have been good.
2. First session discussing research gaps/priorities: clearer expectations/explanation of what was needed; perhaps on option to write notes and discuss, so all thoughts captured despite lack of time.
3. Time constraints - restricted discussions. Perhaps more small discussion groups (3-4 rather than 2)

More detail prior to the workshop

1. I would've liked to have a copy of the material discussed today prior to the day. Didn't have time to read participant's bios in the pack. Would've liked to participate in other (>2) table topic discussions; The day could have been longer (workshop 9-5) or over 2 days; Perhaps some new topics ideas/suggestions could be thought of prior to coming to the workshop
2. more detail prior to today which fills out the topics we were asked to rate - so we could think about it beforehand

Logistics

1. The audio levels @ breakout tables - need separate areas
2. Signage in the carpark

Positive feedback

1. What I have heard and contributed today I have some insights to take back to my role as a consumer representative, so I'm very pleased.
2. I think this was a well co-ordinated and effective day/workshop
3. It would be good to do more of these as a way of capturing face to face discussion groups apart from the surveys

Other

1. Not sure if small sessions picks up key issues
2. Communication between the professional and the patient; more information on different illnesses
3. Synthesised issues- <21
4. The session before lunch was a bit to vague and all over the place; needed a bit more structure/direction about what was needed and purpose of the discussion
5. More Aboriginal voice in the group
6. Sometimes it was hard to get people to focus on identifying an issue rather than solving it

**Question 4: Do you have any other thoughts, comments, reflections or ideas?**

Would like to be informed of, or involved in the prioritised research

1. Looking forward to seeing the outcomes of today and potential for further participation
2. I would like to carry out some research; I think the underlying issues are lack of communication and community. Thanks - fabulous day !
3. Happy to hear that more consumers can assist in future research; good avenue to pool ideas we may have for future research plus be invited to be involved; have this group invited to join the Cochrane Consumers and Communication Group even if it's only as a consumer register to tap into.
4. would like feedback as to the progress and outcomes of the project.
5. Would love to hear about further opportunities to be involved. These session help to support communication across services - policy makers, gov, health profs and consumers - Great!

General positive feedback

1. Great session
2. Great day!
3. Great work by the team; well integrated groups; well run workshop; I will be interested in the issues identified as the higher priorities
4. Liked the breakout small group discussion element. Very good day overall. I hope such engagement days continue in the future.
5. Fantastic Day; Fantastic day!! Facilitator is amazing :) :) :) and the table facilitators were great too. Thank you for this amazing day and for involving consumers and providers.
6. Well planned, prepared workshop
7. I felt the workshop was very well organised. The transport/parking was well communicated. The facilitation was great. The organisation of the segments was good.
8. Thank you. Also, I appreciated the chance to meet other people also interested in this area
9. This has informed my role as a consumer representative, hence I'll be able to contribute more perspective which could add some insight to health practice at [health service] to better cater for health consumers. Cheers.

Suggestions for improvement

1. Expanding the pre-reading with some of the more detailed info may have enabled more reflection and thought prior to the workshop. However overall it has been an interesting, thought provoking day
2. Can you open discussions with more varied groups, especially disadvantaged or isolated, rather than the top-heavy educated/health professional/policy makers. May require personal emails, special interest group discussions (i.e. ESL groups, alternate cultures, LGBTI). Approach within their sphere of comfort, where they feel able to contribute. Would give a much more balanced view.

Other

1. Oral health is an issue that should be high on the health issues. If you are not happy with the way your teeth or gums are your health suffers.
