# Supplementary material for: Evaluation of the Cochrane Consumers and Communication Group’s systematic review priority-setting project
Source: Health Res Policy Syst. 2020 Sep 2;18:98. doi: 10.1186/s12961-020-00604-x (PMC7465879; doi:10.1186/s12961-020-00604-x)
Supplement: Supplementary file 2 — Additional file 2. Evaluation raw data [file 12961_2020_604_MOESM2_ESM.docx]

**Additional file 2 – Evaluation raw data**

From: Synnot et al. Evaluation of the Cochrane Consumers and Communication Group’s systematic review priority setting project

**Table S1. Examples of the process evaluation raw data items used within each question/conceptual element**

| **Element** | **Questions** | **Met?** | **Examples of data considered for each question (*with data source*)** |
| --- | --- | --- | --- |
| Stakeholder engagement | 1. Were key stakeholders who might be affected by the choice of review topics, or seek to use the reviews, (such as consumers, health professionals and decision makers) involved effectively in the decision-making process? | Yes | *Project documents (steering group meeting minutes)*  Steering group (n = 11) included consumers and consumer groups, policymakers, health professionals, health services, and research funders. Examples of how the steering group influenced key decisions include: they changed the scope of the topics being prioritised (from a narrow scope to a broad scope that reflected CCCGs review scope), and it was their suggestion that the final report was co-produced with stakeholders. They also approved the final selection of five priority Cochrane Review topics.  *Communication materials (publication describing the workshop stage (Synnot, Tong et al. 2019))*  The workshop included 28 people, with 50% representing consumers and carers and the remainder working in clinical, managerial, policy or client-focussed health roles. They were involved in decision-making through discussion and voting, reducing the list of priority questions to the top 12. The final stage of the prioritisation involved just the project team and the steering group. |
|  | 1. Were multiple techniques were used to identify stakeholders? | Yes | *Project documents (emails)*  Potential steering group identified through the networks of project team.  *Project documents (steering group minutes)*  In the first meeting, steering group members encouraged face to face recruitment for hard to reach groups, such as older people (subsequently discounted for reasons of resource use). Members subsequently suggested phone calls to organisations that work with people from culturally and linguistically diverse groups and Indigenous people (which was done).  *Communication materials (paper describing online survey stage (Synnot, Bragge et al. 2018))*  Purposive and snowball sampling was conducted for the online survey; mainly online recruitment via emails and newsletters. |
|  | 1. Were stakeholders offered multiple ways to contribute? | Yes | *Project documents (steering group minutes)*  Steering group contributed face-to-face or by phone. Those who couldn't attend meetings were offered a one-on-one follow up.  *Communication materials (paper describing online survey stage (Synnot, Bragge et al. 2018))*  Stakeholders could contribute by being in the steering committee, doing the online survey (including doing it by post or by phone), or taking part in the workshop. |
|  | 1. Was CCCG committed to genuine engagement through partnership and empowerment? | Yes | *Communication materials (Paper describing online survey stage (Synnot, Bragge et al. 2018))*  "We worked in partnership with consumers and other stakeholders to plan and undertake all project stages. Our approach was informed by the principles of coproduction, that is, recognising expertise, building on strengths, enabling shared control and mutually beneficial and supported relationships."  *Project documents (Workshop running sheet)*  Examples of the some of the ways CCCG demonstrated partnership and empowerment at the workshop: ensuring 50% of attendees were consumers or carers to mitigate power imbalances, providing taxi vouchers for those who needed them, providing $50 voucher for consumers/carers and those who took a day off work to attend and seeking short bios for all participants to describe themselves and the perspective they brought to the day. |
|  | 1. Were stakeholders satisfied with their level of involvement in the decision-making process? | Yes | Not specifically surveyed but can be implied.  *Project documents (steering group meeting minutes)*  Steering group members endorsed their proposed role in the project at the first meeting.  *Workshop feedback survey*  All workshop participants (25/25) agreed with the statements 'I feel that my contribution was valued and heard’, and 'the materials and resources used during the day helped me understand my role and make a contribution'. |
| Use of explicit process | 1. Was the priority setting process pre-determined and made transparent to stakeholders? | Yes | *Project documents (steering group meeting minutes)*  Project plan was provided on the project webpage at the outset of the project. Shared with steering group at the first meeting.  *Project documents (online survey recruitment materials)*  Recruitment materials and online survey information sheet linked to project page and provided brief description of subsequent project stages |
|  | 1. Were internal *and* external stakeholders probed for information relevant to priority setting decisions? | Yes | Probing internal stakeholders was not applicable as we specifically sought to probe external stakeholders. All stakeholders involved in the steering group, online survey and the workshop were external to the project team/CCCG. |
|  | 1. Were the methods used to set priorities understandable, transparent and relevant for different stakeholders? | Yes | *Project documents (steering group meeting minutes)*  Steering group reviewed and approved the methods used at each step.  *Communication materials (final report (Synnot 2016))*  Quote provided by a participant for the final report: “Thank you so much for all your help to allow me to participate in the workshop. It was one of the best experiences of my life, I felt ‘heard’ and I hope I was able to help in some way.’ |
|  | 1. Was communication with stakeholders well-coordinated, systematic and well-planned? | Yes | *Project documents (steering group meeting minutes)*  Steering group meetings had agenda, with pre-reading and minutes circulated quickly  *Communication materials (paper describing online survey stage (Synnot, Bragge et al. 2018))*  The online survey was distributed to approximately 1000 email addresses with weekly reminders sent while the survey was open. |
|  | 1. Was information about the project communicated effectively using multiple vehicles? | Yes | *Communication materials (project webpage (Centre for Health Communication and Participation 2019))*  A project webpage was created at the project outset, describing the stages of the project, and then providing updates and results as the project progressed.  *Communication materials (final report (Synnot 2016))*  Final report was coproduced with stakeholders and used text and infographics. |
| Information management | 1. Was information used to set priorities, including sources used and how it was collected and collated, made explicit to those setting priorities? Was it deemed sufficient? | Partial | *Project documents (workshop pre-reading materials)*  The information/sources used to set priorities was made explicit to stakeholders in workshop pre-reading and on the day where the priorities to that point had come from.  *Project documents (steering group meeting minutes)*  The group endorsed the processed used to derive the priority Cochrane Review topics, and felt the process was clear and easy to follow. The steering group did not get a say in the final decision-making step, but endorsed the approach we used. |
| Consideration of values and context | 1. Was the mission, vision and values of CCCG used to guide priority setting decisions, and made explicit? | Yes | *Centre 2019 Year in Review Report (Centre for Health Communication and Participation 2019)*  Mission: “The Centre for Health Communication and Participation conducts research to improve people’s health and wellbeing through the generation and promotion of evidence-informed strategies for consumer communication and participation in health. We aim to strengthen the active involvement of consumers in health care, policy and research.” |
|  | 1. Did priority setting decisions take into account CCCG’s strategic directions as a unit? | Yes | *Centre 2019 Year in Review Report (Centre for Health Communication and Participation 2019)*  Two of the Centre’s strategic directions:  -“To make the Centre integral to the landscape of consumer participation and communication in health through strategic partnerships with researchers, policy makers, funders and consumers.  -To share and promote the work we do to create a cohesive platform of consumer participation and communication evidence which is of high-quality and accessible to a range of audiences.” |
|  | 1. Were the (potential) values of stakeholders, both those involved in the project and those not involved, were used to guide priority setting decisions? | Yes | *Communication materials (Publication describing the workshop stage (Synnot, Tong et al. 2019)*  One of the editorial criteria used for selecting the top five priority reviews, “The author team is agreeable to formally including consumers and/or other stakeholders in their review planning, conduct and/or dissemination” reflected one of the top priorities at the workshop (“Health services do not properly involve consumers and carers in health service planning and design”). |
| Revision or appeals mechanism | 1. Was there a formal mechanism for reviewing decisions, whereby stakeholders could identify failures and errors or contribute new information? | Partial | No formal appeals mechanism was used one the final priorities were set, however:  *Project documents (steering group meeting minutes)* Steering group members could review and suggest changes to project direction (for example they suggested additional recruitment efforts to increase the diversity of participants).  *Communication materials (Publication describing the workshop stage (Synnot, Tong et al. 2019)*  At the workshop, participants were first invited to review and add to, the list of priorities generated in the online survey. As a result, one new priority was added to the list of priorities for voting on later in the day. |

**Table S2. Examples of the outcome evaluation raw data items used within each question/conceptual element**

| **Element** | **Questions** | **Met?** | **Examples of data considered for each question (*with data source*)** |
| --- | --- | --- | --- |
| Improved stakeholder understanding | 1. Did stakeholders obtain more than a knowledge of the priority setting process, but gained insight into broader aspects of priority setting (e.g. the rationale for priority setting generally) and/or the CCCG/Cochrane (e.g. its mission, values)? | Yes | *CCCG Editorial team reflections*  One steering group member initiated a priority setting exercise in her workplace and another became a co-author on one of the priority reviews.  *Workshop feedback survey*  25/25 workshop attendees agreed with the statements, “The information I received before coming helped me understand what was expected of me before I came” and “The materials and resources used during the day helped me understand my role and make a contribution”. |
| Shifted priorities and/or reallocation of resources | 1. Were more Cochrane Reviews topics selected that were relevant to stakeholders? | Yes | *Communication materials (publication describing the workshop stage (Synnot, Tong et al. 2019))*  The five priority Cochrane reviews selected closely reflected priority topics selected by stakeholders (e.g. the top-ranked priority was “the term patient-centred care is poorly understood and implemented by health services and health professionals” and one of the priority reviews selected was (at the time) titled, “Interventions for providers to promote a patient-centred approach in clinical consultations”). |
|  | 1. Did Cochrane Review topics that reflect the priorities of stakeholders get funded and conducted? | Partial | *CCCG Editorial team reflections*  -Two reviews were funded. All reviews are underway (and all have published protocols) but none yet published.  -Ways that CCCG is supporting these reviews: reallocated editorial resources such as providing rapid assistance with methodological queries and prioritising search development. |
| Improved decision-making quality | 1. Over time, were CCCG decisions and strategic direction more consistent with the priorities generated? | Yes | *CCCG Editorial documents for review production*  From the Title Proposal Form for CCCG authors: “We have also undertaken a project to explore consumers’, researchers’ and health policy makers’ priorities for Consumers and Communication reviews. (…) Preference will be given to title proposals that reflect one or more of these priority areas.”  *CCCG Editorial team reflections*  Three editorial staff involved in the priority setting and/or coproduction activities of the Centre has led to the initiation of a coproduction network for Australian researchers and consumers, and two new collaborations with international researchers involved in stakeholder engagement activities. |
|  | 1. Did future CCCG priority setting activities aim to build on earlier efforts? | N/A | Question not able to be answered at the time of the evaluation. |
| Stakeholder acceptance and satisfaction | 1. Did stakeholders express satisfaction with the process? | Yes | *Workshop feedback survey*  Workshop participants gave mostly positive feedback: 14/25 praised the structure and/or facilitation of the day. Several called the day ‘fantastic’ and ‘great’.  *CCCG Editorial team reflections*  Five workshop participants and steering group members subsequently joined author teams or stakeholder panels for the priority reviews. |
|  | 1. Did stakeholders partner with researchers to conduct the priority Cochrane Reviews? | Yes | *CCCG Editorial team reflections*  -All priority reviews involve stakeholders, for example as co-authors or members of a stakeholder panel.  -A group of health policymakers (not involved in the priority setting) provided peer review of the protocol of one of the priority reviews. |
|  | 1. Did stakeholders use the results of the priority reviews? | N/A | Question not able to be answered at the time of the evaluation. |
| Positive externalities | 1. Were the results of the priority setting process were shared widely | Yes | *Project documents*  Results of the priority setting project were distributed to all stakeholders who took part via a final report, promoted in relevant newsletters, blogs, policy maker seminars and in academic presentations and publications. |
|  | 1. Research funders and research institutes included the priorities as part of their research agenda or strategic planning | No | No evidence found to support this. |
|  | 1. The priority setting process and/or its results was emulated by or influenced the work of other organisations | Yes | *CCCG Editorial team reflections*  -Methods were replicated by Safer Care Victoria (government department) to develop their policy on consumer engagement in healthcare (Horvat 2019).  -A systematic review team cited our findings to provide justification for their systematic review Selman (2017). |
|  | 1. Did the priority reviews result in changed policies, legislation or clinical practice? | N/A | Question not able to be answered at the time of the evaluation. |

**References**

Centre for Health Communication and Participation. (2019). "Research Priority Setting." Retrieved 4 March 2020, from <https://www.latrobe.edu.au/chcp/projects/research-priority-setting>.

Centre for Health Communication and Participation (2019). Year in Review 2018, La Trobe University, Victoria.

Horvat, L. (2019). Partnering in healthcare: A framework for better care and outcomes. Melbourne, Safer Care Victoria, State Government of Victoria.

Selman, L. E., L. J. Brighton, A. Hawkins, C. McDonald, S. O'Brien, V. Robinson, S. A. Khan, R. George, C. Ramsenthaler, I. J. Higginson and J. Koffman (2017). "The Effect of Communication Skills Training for Generalist Palliative Care Providers on Patient-Reported Outcomes and Clinician Behaviors: A Systematic Review and Meta-analysis." Journal of Pain and Symptom Management **54**(3): 404-416.e405.

Synnot, A. (2016). Stakeholder priorities for research in health communication and participation: Findings from the Cochrane Consumers and Communication priority setting project. Melbourne, Centre for Health Communication and Participation, La Trobe University.

Synnot, A., P. Bragge, D. Lowe, J. S. Nunn, M. O’Sullivan, L. Horvat, A. Tong, D. Kay, D. Ghersi, S. McDonald, N. Poole, N. Bourke, N. Lannin, D. Vadasz, S. Oliver, K. Carey and S. J. Hill (2018). "Research priorities in health communication and participation: international survey of consumers and other stakeholders." BMJ Open **8**(5): e019481.

Synnot, A. J., A. Tong, P. Bragge, D. Lowe, J. S. Nunn, M. O’Sullivan, L. Horvat, D. Kay, D. Ghersi, S. McDonald, N. Poole, N. Bourke, N. A. Lannin, D. Vadasz, S. Oliver, K. Carey and S. J. Hill (2019). "Selecting, refining and identifying priority Cochrane Reviews in health communication and participation in partnership with consumers and other stakeholders." Health Research Policy and Systems **17**(1): 45.
